# Somatic support cells regulate germ cell survival through the Baz/aPKC/Par6 complex

**DOI:** 10.1242/dev.169342

**Published:** 2019-04-15

**Authors:** Susanna E. Brantley, Margaret T. Fuller

**Affiliations:** Department of Developmental Biology, Stanford University School of Medicine, Stanford, CA 94305, USA

**Keywords:** *Drosophila*, Spermatogenesis, Cyst cells, Par complex, JNK

## Abstract

Local signals and structural support from the surrounding cellular microenvironment play key roles in directing development in both embryonic organs and adult tissues. In *Drosophila*, male germ cells are intimately associated and co-differentiate with supporting somatic cells. Here, we show that the function of the Baz/aPKC/Par6 apical polarity complex in somatic cyst cells is required stage specifically for survival of the germ cells they enclose. Although spermatogonia enclosed by cyst cells in which the function of the Par complex had been knocked down survived and proliferated, newly formed spermatocytes enclosed by cyst cells lacking Par complex proteins died soon after onset of meiotic prophase. Loss of Par complex function resulted in stage-specific overactivation of the Jun-kinase (JNK) pathway in cyst cells. Knocking down expression of JNK pathway components or the GTPase Rab35 in cyst cells lacking Par complex function rescued the survival of neighboring spermatocytes, suggesting that action of the apical polarity complex ensures germ cell survival by preventing JNK pathway activation, and that the mechanism by which cyst cells lacking Par complex function kill neighboring spermatocytes requires intracellular trafficking in somatic cyst cells.

## Abstract (precis)

**Summary:** The Par polarity complex suppresses JNK pathway activity in *Drosophila* somatic support cells to allow stage-specific germ cell survival.

## INTRODUCTION

Understanding how local interactions between epithelia and their non-epithelial neighbors shape cell fate specification, proliferation, differentiation and survival is crucial for understanding how organs develop and how adult stem cell systems maintain and restore tissues. Many organs comprise sheets of columnar epithelial cells partnered with structurally and functionally distinct mesenchyme, often derived from a different germ layer. Proper development and function of such organs requires interaction and signaling from mesenchymal cells to their epithelial neighbors (Grobstein, 1967), as well as the reverse: cells in epithelia have been shown to act as signaling centers for tissue morphogenesis in the vertebrate limb bud, the fly wing and several other organ systems (Niswander et al., 1993; Gibson and Schubiger, 2000; Pallavi and Shashidhara, 2005; Frank and Rushlow, 1996).

Gamete differentiation in *Drosophila* provides powerful models for studying how interactions between epithelial-like somatic support cells and the non-epithelial germ cells they enclose regulate co-differentiation of somatic and germ cells within germ cell cysts. In ovaries, columnar and squamous epithelial cells of somatic stem cell origin encase germline-derived nurse cells and the oocyte (Wu et al., 2008). The somatic epithelial cells help direct polarity and differentiation of the developing oocyte (Gonzales-Reyes and St Johnson, 1998; Godt and Tepass, 1998) and regulate nurse cell death (Giorgi and Deri, 1976). In *Drosophila* testes, the functional unit of differentiation is the cyst, in which two somatic cyst cells encapsulate and co-differentiate with a clone of germ cells (Gönczy and DiNardo, 1996; Fuller, 1993). The two somatic cells form occluding and adherens junctions with each other, much like epithelial cells in other tissues, sealing the cyst (Fairchild et al., 2015; Smendziuk et al., 2015).

Here, we show that the function of apical polarity complex proteins is required in the epithelial-like somatic cyst cells of the *Drosophila* testis to ensure stage-specific survival of the male germ cells they enclose. Epithelia are composed of polarized cells that establish apical and basal domains of the plasma membrane and maintain connections with neighbors in the epithelium such that the cell polarity is echoed across the plane of the multicellular sheet. Across metazoans, apical domains of polarized epithelial cells are established and maintained through action of an apical polarity complex composed of the core components Bazooka (Par3), Par6 and aPKC, which are conserved from *C. elegans* to man (Baum and Georgiou, 2011). We show that function of the Par complex is required in somatic cyst cells to restrict activation of the Jun kinase (JNK) signaling pathway. In the absence of this protection, loss of apical polarity complex function in cyst cells results in stage-specific, non-autonomous cell death of neighboring germ cells. Death of the spermatocytes is dependent on function in cyst cells of the recycling endosome small GTPase Rab35, which is reminiscent of how stretch follicle cells promote death of nurse cells in maturing eggs chambers in the ovary (Timmons et al., 2016).

## RESULTS

### Par complex function is required in cyst cells for survival of early spermatocytes

**Fig. 1.**
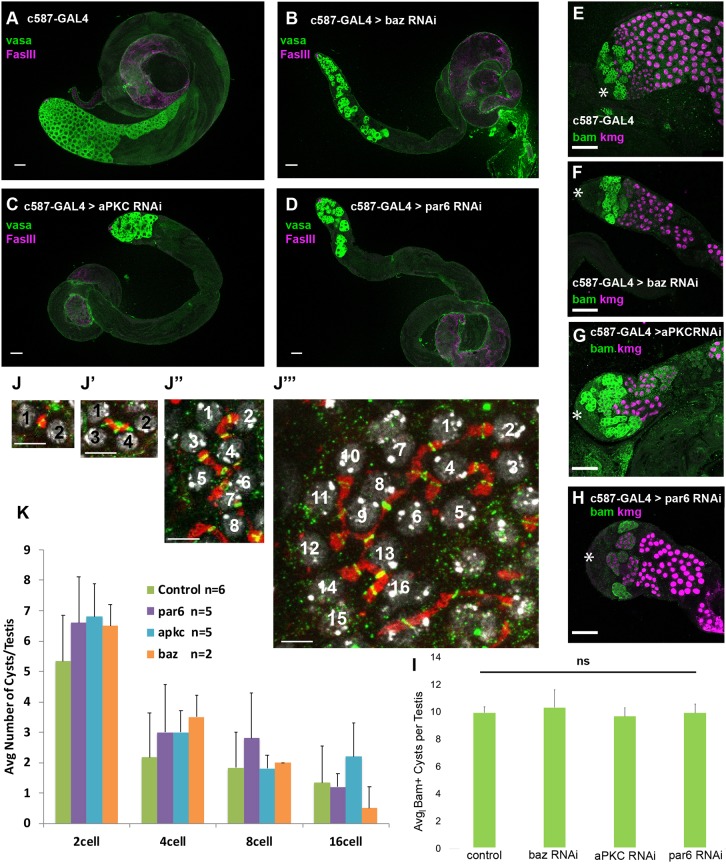
**The Par complex is required in somatic cyst cells for germ cell survival.** (A-D) Immunofluorescence images of testes from flies 6 days after a shift to 30°C stained using anti-Vasa (green, germ cells) and anti-FasIII (magenta, hub). (E-H) Immunofluorescence images of testes from flies shifted to 30°C for 8 days after eclosion stained using anti-Bam (green, spermatogonia) and anti-Kmg (magenta, spermatocyte nuclei) antibodies. Asterisks indicate the apical hub. Scale bars: 50 μm. (I) Average number of Bam-positive cysts per testis (from experiment in E-H). Data are mean±s.e.m. (J-J‴) Immunofluorescence staining using anti-Spectrin (fusome, red) and anti-phosphoTyr (ring canal, green) antibodies, showing DNA (DAPI, gray). These images were used to determine spermatogonial number per cyst. Scale bars: 15 µm. (K) Quantification of spermatogonial cyst type per testis 6 days after a shift to 30°C. Significance was determined using a two-tailed Student's *t*-test in I and K. ns, not significant. Data are mean±s.e.m.

Loss of function of the Par complex components aPKC, Par6 or Bazooka (Baz/Par3) in cyst cells induced by cell type-specific RNAi resulted in stage-specific germ cell loss, occurring soon after germ cells exit mitosis and begin to differentiate as early spermatocytes. RNAi constructs against *aPKC*, *par-6* or *baz* were expressed in cyst cells under the control of c587-GAL4, which drives expression in the somatic cyst cell lineage (Decotto and Spradling, 2005). To prevent lethality due to GAL4 activity in somatic cells during developmental stages, the flies also carried a transgene encoding a temperature-sensitive GAL80^ts^ allele in the genetic background to repress hairpin production at the permissive temperature (22°C). Flies were raised to adulthood at 22°C, shifted to 30°C at eclosion to allow expression of the RNAi hairpins, maintained at 30°C and the effect on testes was scored at different time points after the shift (Fig. S1). In control males subject to this temperature regimen, plentiful germ cells were visible after immunofluorescence staining of testes with anti-Vasa as small spermatogonia near the testis apical tip and progressively larger spermatocytes starting several cell diameters away from the apical tip of the testis (Fig. 1A, diagrammed in Fig. S2A). Loss of Baz, aPKC or Par6 in cyst cells under conditions of acute knockdown led to progressive loss of large Vasa-positive spermatocytes, with the majority of mature spermatocytes no longer present by day 6 of knockdown (Fig. 1A-D and Fig. S1).

Notably, the number of spermatogonial cysts was unaffected by knockdown of Par complex components in cyst cells. Immunofluorescence staining for the mid-to-late spermatogonial marker Bam showed that there was no significant change in the number of Bam-positive cysts per testis following knockdown of Par-complex function in cyst cells compared with controls (Fig. 1E-I). Likewise, counts based on staining for the fusome and ring canals that connect mitotic sister germ cells within a cyst showed no difference in the distribution of 2-, 4-, 8- or early 16-cell germ cell cysts following Par complex knockdown in cyst cells (Fig. 1J,K).

Germ cells in testes where Par complex components had been knocked down in cyst cells did appear to successfully switch from spermatogonial to spermatocyte state. Immunofluorescence staining revealed that some germ cells remaining after 6 days of knockdown lacked Bam protein and expressed the spermatocyte-specific transcriptional regulator Kmg (Fig. 1E-H) and the spermatocyte-specific translational regulator Rbp4 (Fig. S1), although the number of such spermatocyte cysts observed was far fewer than in control testes.

**Fig. 2.**
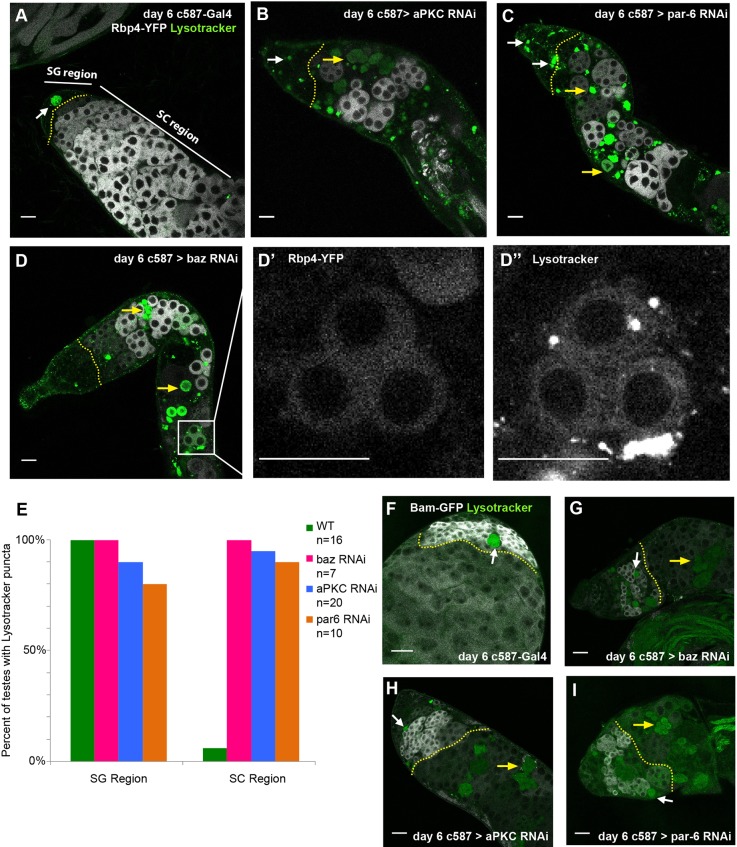
**Loss of Par complex components in cyst cells leads to non-autonomous cell death in early spermatocytes.** (A-D) Live imaging of testes from flies expressing Rbp4-eYFP (white) 6 days after a shift to 30°C incubated with Lysotracker (green). Yellow dotted lines indicate the boundary between the spermatogonial (SG) and spermatocyte (SC) regions of the testis (white arrow, SG Lysotracker signal; yellow arrow, SC Lysotracker signal). (D′,D″) A group of dying spermatocytes that co-stain for Rbp4-eYFP and Lysotracker. Scale bars: 25 μm in A-D″. (E) Percentage of testes with Lysotracker signal in the SG or SC region (*n*>10 testes per genotype). (F-I) Live imaging of testes from flies expressing *bam*-GFP (white) 6 days after shift to 30°C and incubated with Lysotracker (green). Dotted yellow line indicates the boundary between the SG and SC regions of the testis (white arrow, SG Lysotracker signal; yellow arrow, SC Lysotracker signal). Scale bars: 25 μm in F-I.

The non-autonomous loss of germ cells following knockdown of Par complex function in cyst cells was due to death of germ cells in the spermatocyte region of the testis. Staining with LysoTracker to mark acidified dying cells confirmed that germ cells in control testes occasionally die, as has been reported previously (Yacobi-Sharon et al., 2013). However, this normal germ cell death primarily affected spermatogonial cysts, rather than spermatocytes. In contrast to controls (Fig. 2A), a significantly higher percentage of testes in which Par complex function had been knocked down in cyst cells showed LysoTracker staining among germ cells that express the early spermatocyte marker Rbp4 (Fig. 2B-D, quantified in E). Similarly, in testes expressing a GFP-tagged Bam transgene, LysoTracker signal was observed within the Bam-positive region in both control testes (Fig. 2F) and testes in which Par complex function had been knocked down in cyst cells (Fig. 2G-I). However, only in knockdown testes was LysoTracker signal observed farther down the testes in regions in which expression of Bam protein had reduced (Fig. 2G-I).

### Loss of Par complex function in cyst cells did not abolish the somatic permeability barrier

**Fig. 3.**
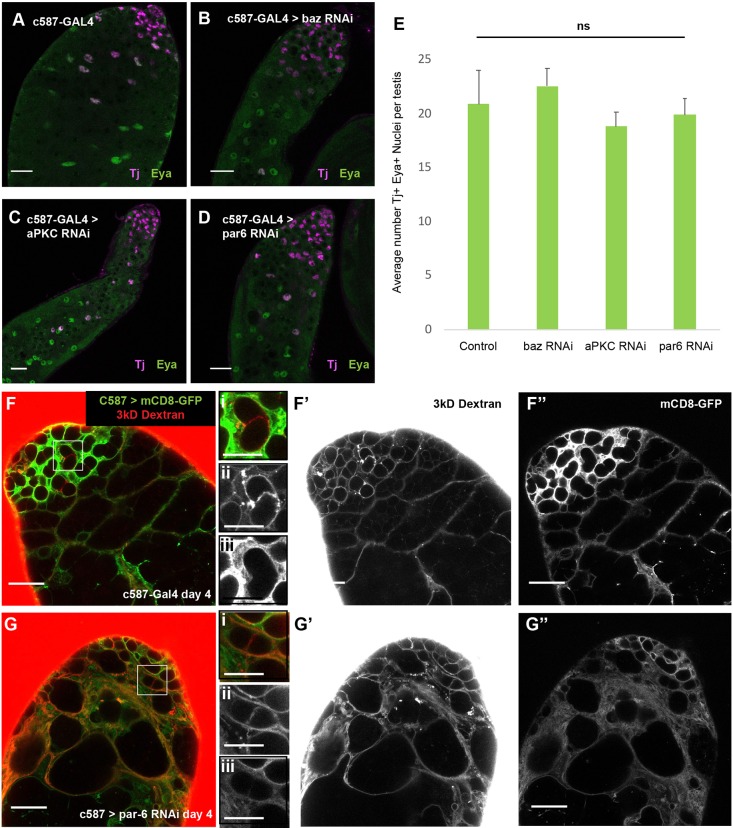
**Cyst cells are not lost and maintain a permeability barrier following Par complex knockdown.** (A-D) Immunofluorescence images of testes from flies shifted to 30°C for 6 days stained using anti-Tj (magenta, early cyst cell nuclei) and anti-Eya (green, late cyst cell nuclei) antibodies. Scale bars: 25 μm in A-D. (E) Average number of Tj and Eya positive nuclei from the experiment in A-D. Significance determined by two-tailed Student's *t*-test. Data are mean±s.e.m. ns, not significant. (F-G″) Images from permeability assays on live testes dissected from flies 4 days after shift to 30°C. 3 kDa Dextran (red in F and G; F′ and G′); mCD8-GFP (green, cyst cell membranes in F and G; F″ and G″). Scale bars: 25 μm in F-G″). Panels on the right of F and G (i-iii) show higher magnification views of indicated areas in F and G (scale bars: 12.5 μm).

Cyst cells appeared to survive and differentiate even when the function of Par complex proteins had been knocked down. Cyst stem cells and their daughters express the nuclear marker Tj throughout the spermatogonial stages of germ cell differentiation. As cyst cells mature, nuclear Eya protein accumulates, such that cyst cells that enclose 16-cell spermatogonia and early spermatocyte cysts usually express both Tj and Eya protein localized to the nucleus. Counts of cyst cell nuclei that stained positively for both Tj and Eya revealed no significant difference in the number of cyst cell nuclei in Par knockdown versus control testes (Fig. 3A-E).

The two somatic cyst cells that surround a germ cell cyst establish discrete junctional domains (Fig. S2B-D) (Fairchild, et al., 2017). Live imaging of isolated spermatocyte cysts from testes that express endogenously tagged Baz::GFP showed this Par complex component concentrated in a distinct belt around the cyst (Fig. S2B), presumably where the two cyst cells meet. Immunofluorescence images of spermatocyte cysts from flies that carry a transgene expressing *Drosophila* E-cadherin [encoded by *shotgun* (*shg*)] tagged with the fluorescent protein Tomato (Shg::Tomato), showed that the cyst cell-cyst cell junction has typical apicobasal polarity domains, in which the Par complex and adherens junction proteins colocalize (Fig. S2C), whereas the basolateral domain protein Dlg was juxtaposed to but did not colocalize with Shg and Baz (Fig. S2D).

**Fig. 4.**
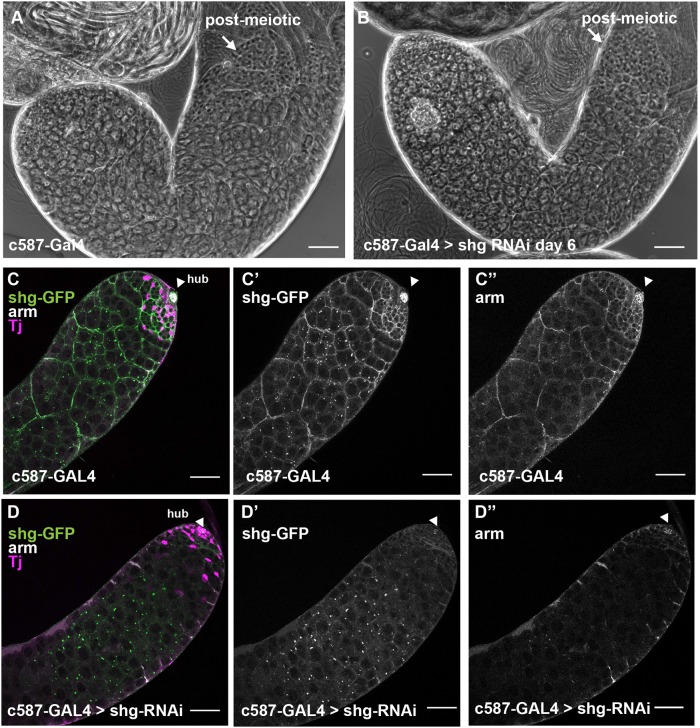
**Cyst cells do not require adherens junctions to maintain normal testis morphology.** (A,B) Phase-contrast images of unfixed testes from (A) control *c587-GAL4* and (B) *shg-RNAi* flies 6 days after a shift to 30°C. Scale bars: 50 μm. (C-D″) Immunofluorescence images of testes from flies expressing shg-GFP 6 days after shift to 30°C stained using anti-GFP (C,D,C′,D′), anti-Arm (C,D,C″,D″; white) and anti-Tj (magenta, cyst cell nuclei) antibodies. Scale bars: 50 μm. Arrowheads indicate the hub.

The stage-specific death of spermatocytes following Par complex knockdown in cyst cells did not appear to be due to a lack of formation of cell-cell junctions between cyst cells. The permeability barrier normally established by septate junctions between encapsulating cyst cells around four-cell spermatogonia (Fairchild et al., 2015) appeared to be formed even when Par complex function had been knocked down in cyst cells. Fluorescently conjugated 3 kDa Dextran dye, which can infiltrate around germline stem cells, gonialblasts and the germ cells within two-cell spermatogonial cysts, is normally excluded from passing the cyst cell barrier once a septate junction is established between the two encapsulating cyst cells at the four-cell stage and beyond. Although the dye was able to infiltrate early germ cell cysts in both control and knockdown testes, the 3 kDa Dextran was excluded from the late spermatogonial and early spermatocyte cysts that remain in the testis after knockdown of Par complex proteins in cyst cells (Fig. 3F-G; Fig. S3A-C). In addition, loss of function of the adherens junction component *Drosophila* E-Cadherin (*shg*) in cyst cells did not phenocopy the spermatocyte death phenotype caused by loss of function of Par-complex components, suggesting that the germ cell death is not due to failure to make adherens junctions. Testes appeared normal under phase-contrast microscopy following knockdown of *shg* in somatic cyst cells (Fig. 4A,B). Staining of testes from flies expressing a GFP-tagged Shg protein confirmed that the knockdown was effective at reducing levels of GFP to below detection by immunofluorescence and also decreased levels of the *Drosophila* β-catenin protein, Armadillo, at cyst cell junctions (Fig. 4C,D).

### Loss of Par complex function activates the JNK pathway in cyst cells to cause germ cell death

**Fig. 5.**
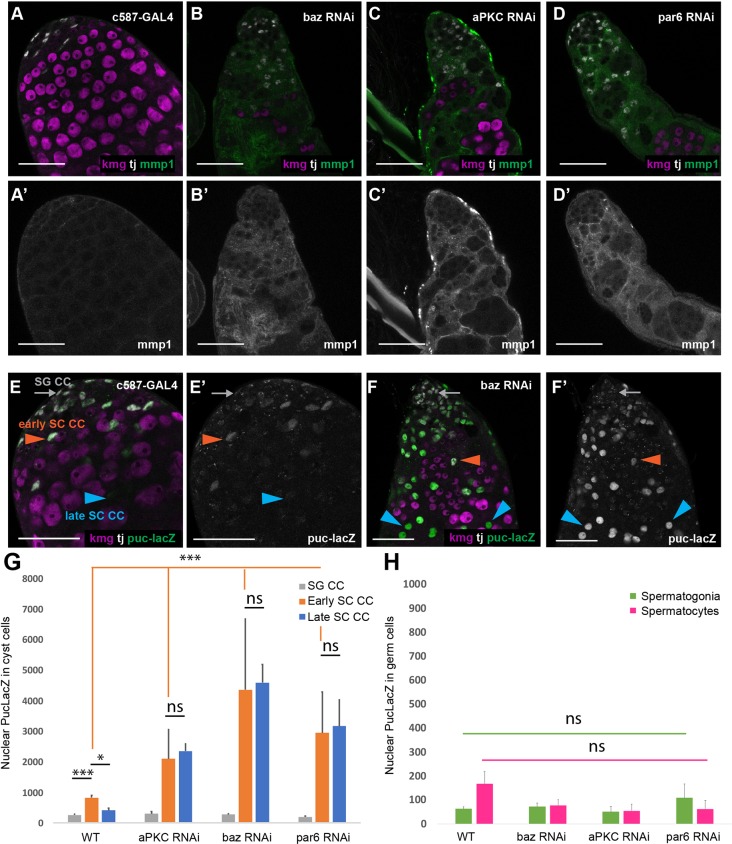
**The JNK signaling pathway is activated in spermatocyte-associated cyst cells following Par complex knockdown.** (A-D′) Immunofluorescence images of testes from flies shifted to 30°C for 6 days after eclosion and stained using anti-Mmp1 (green; A′-D′), anti-Tj (magenta, cyst cell nuclei; A-D) and anti-Kmg (red, spermatocyte nuclei; A-D) antibodies. (E-F′) Immunofluorescence images of testes from Puc-LacZ flies shifted to 30°C for 6 days after eclosion and stained using anti-β-gal (E-F′; green), anti-Tj (white, cyst cell nuclei; E,F) and anti-Kmg (magenta, spermatocyte nuclei; E,F) antibodies. SG CC (gray arrows), spermatogonia-associated cyst cell; early SC CC (orange arrowheads), early spermatocyte-associated cyst cell; late SC CC (blue arrowheads), late spermatocyte-associated cyst cell. Scale bars: 50 μm. (G) Quantification of nuclear Puc-LacZ fluorescence intensity from experiments in E and F. Significance is determined using a two-tailed Student's *t*-test. (***P<10^−8^ compared with control; *P<10^−3^ compared with control). Data are mean±s.e.m. *n*>50 cells per genotype from each of three biological replicates. (H) Quantification of nuclear Puc-LacZ fluorescence intensity from experiment in E-F′. Significance was determined using a two-tailed Student's *t*-test. Data are mean±s.e.m. *n*>30 cells per genotype from each of three biological replicates.

Knockdown of Par-complex components resulted in increased expression of both the JNK target gene Mmp1 (Fig. 5A-D) and the JNK pathway transcriptional reporter Puc-LacZ in cyst cells (Fig. 5E-F). In control testes, Mmp1 was barely detectable by immunofluorescence staining (Fig. 5A). Following loss of Par complex function in cyst cells, Mmp1 staining was detected in the cytoplasm of cyst cells, although not at the apical tip of the testis (Fig. 5B-D). Likewise, Puc-LacZ was also significantly upregulated in cyst cells associated with spermatocytes when function of the Par complex was knocked down. In control testes (Fig. 5E), expression of Puc-LacZ was slightly higher in nuclei of Tj-positive cyst cells that neighbor Kmg-positive early spermatocytes (early SC CCs) compared with Tj-positive cyst cells that neighbor only Kmg-negative spermatogonia (SG CCs), and expression of Puc-LacZ was significantly lower in more mature Tj-negative spermatocyte associated cyst cells (late SC CCs). When function of Par complex components was knocked down in cyst cells, the level of Puc-LacZ detected was significantly increased in nuclei of cyst cells associated with early spermatocytes (early SC CCs) but not in cyst cell nuclei that neighbor spermatogonia (SG CCs) (Fig. 5E-G). Puc-LacZ levels remained high in the persisting cyst cell nuclei that no longer express Tj (late SC CCs) (Fig. 5F,G). Notably, germ cells did not show an increase in markers of JNK pathway activity following knockdown of Par complex proteins in cyst cells (Fig. 5H). Together, these data raised the possibility that stage-specific hyperactivation of the JNK pathway in somatic cyst cells might trigger events leading to non-autonomous death of the germ cells within the cyst.

**Fig. 6.**
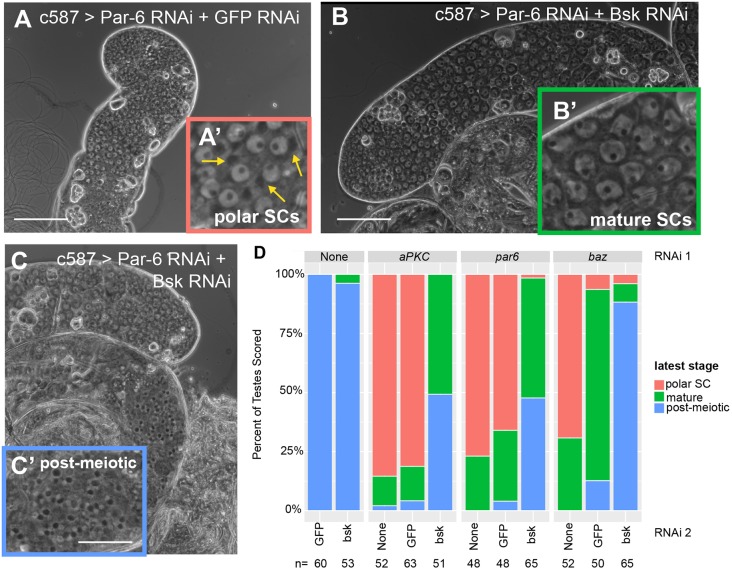
**The Par complex restricts JNK signaling in cyst cells to prevent spermatocyte death.** (A-C) Phase-contrast images of unfixed testes showing representative phenotypes from *c587-GAL4*>UAS-*par6* RNAi;UAS-*gfp* RNAi flies (A) and *c587-GAL4*>UAS-*par6* RNAi;UAS-*bsk* RNAi flies (B,C). Insets show higher magnification images of key germ cell stage. (A′) Young polar spermatocytes (yellow arrows indicate polarized mitochondrial derivative); (B′) mature spermatocytes; (C′) post-meiotic onion stage spermatids. Scale bars: 100 μm. (D) Quantification of phenotype distribution for testes of the indicated genotypes 8 days after a shift to 30°C. *P*<10^−12^ by Fisher's exact test compared with double RNAi control.

**Fig. 7.**
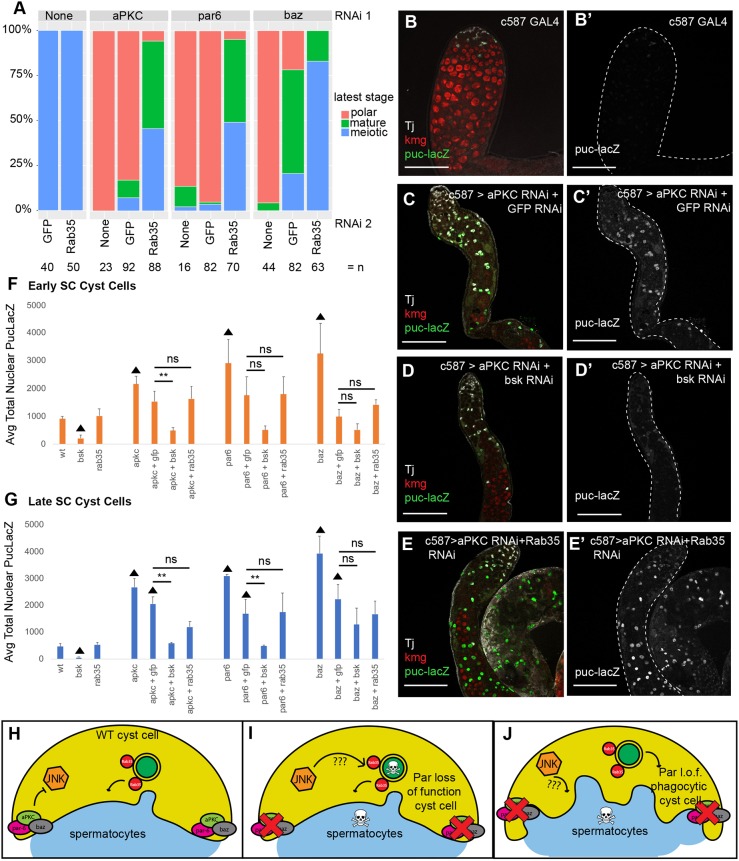
**Rab35 acts downstream or in parallel to the JNK pathway in cyst cells following Par complex loss of function to cause spermatocyte death.** (A) Quantification of phenotype distribution for testes of the indicated genotypes 8 days after a shift to 30°C. *P*<10^−12^ by Fisher's exact test compared with double RNAi control. (B-E′) Immunofluorescence images of testes from flies expressing Puc-LacZ (green, B-E; gray, B′-E′) shifted to 30°C for 8 days after eclosion and stained using anti-βgal (green), anti-Tj (gray, cyst cell nuclei) and anti-Kmg (red, spermatocyte nuclei) antibodies. Scale bars: 50 μm. (F,G) Quantification of nuclear Puc-LacZ fluorescence intensity in (F) early and (G) late spermatocyte-associated cyst cells (SC CCs). *n*>25 cells from each of at least two biological replicates. Significance was determined using Student's one-tailed *t*-test (**P<0.05; ▴ indicates *P*<0.05 compared with wild type). (H) The Par complex represses JNK pathway activation in wild-type cyst cells. (I,J) Loss of function of the Par complex in cyst cells results in spermatocyte death by activation of the JNK pathway (I), which may deliver a pro-death or pro-apoptotic signal to the germ line via Rab35. (J) Rab35 may also work in parallel to the JNK signaling pathway in Par loss-of-function cyst cells to induce spermatocyte phagocytosis by the cyst cells.

Consistent with the model that activation of the JNK pathway in somatic cyst cells was responsible for the death of early spermatocytes, function of the Jun kinase gene *basket* (*bsk*) in somatic cells was required for the early spermatocyte death following knockdown of Par complex function in cyst cells. Double knockdown of *bsk* along with individual Par complex components partially rescued survival of germ cells compared with controls in which Par complex RNAi hairpins were expressed along with a control RNAi hairpin against GFP. Testes were classified using phase-contrast microscopy of unfixed live squash preparations using the latest germ cell stage observed. Testes with the most extreme phenotype only contained germ cell stages up to polar spermatocytes, an early stage of spermatocyte differentiation that can be identified by a phase-dark cluster of mitochondria at one side of the spermatocyte nucleus (Fig. 6A). Testes scored as having an intermediate phenotype contained, as the latest stage, large mature spermatocytes that no longer had a polarized cluster of mitochondria and had grown in size but not yet undergone meiotic division (Fig. 6B). Testes with the mildest phenotype contained all germ cell stages up to and including early post-meiotic (onion stage) spermatids, which could be identified by the presence of a phase-dark mitochondrial derivative paired with a phase-light small round nucleus in spermatids (Fig. 6C). 69-85% of testes in which Par complex components had been knocked down contained only young polar spermatocytes, with most of the remaining testes scored showing mature spermatocytes but no post-meiotic cysts (Fig. 6D). Adding a second control RNAi line (GFP RNAi) increased the percentage of testes with mature spermatocytes slightly for *aPKC* and *par6*, and more-so for *baz*, and some (4.0% - 12.7%) from double RNAi control males had post-meiotic cells (Fig. 6D). Quantitative rtPCR measuring Par complex transcript levels from whole testes showed that the partial rescue of the *baz* RNAi by adding a second control RNAi construct was due to less efficient knockdown of *baz* under these conditions (Fig. S4A). However, when the second RNAi was against *bsk*, the early spermatocyte death phenotype was considerably more strongly rescued compared with when the second RNAi was against GFP, such that almost all testes scored (96.1-100%) contained either all germ cell stages up through mature spermatocytes or all stages through post-meiotic onion stage germ cells (Fig. 6D). The *bsk* RNAi construct used was efficient at reducing expression of the JNK pathway Puc-LacZ nuclear reporter, confirming that the elevated expression of Puc-LacZ observed with loss of Par complex function in cyst cells was indeed due to increased Jun kinase activity (Fig. 7D,F,G). Knockdown of *baz* or *aPKC* together with *bsk* was equally or more efficient compared with the double knockdown controls as measured by qrtPCR (Fig. S4A-B). Adding *bsk* RNAi with *par-6* RNAi slightly reduced knockdown efficiency of *par-6* compared with the double RNAi control, although levels of *par-6* RNA were still reduced relative to levels in wild-type testes under these conditions (Fig. S4C).

### Function of the recycling endosome GTPase Rab35 is required in cyst cells for spermatocyte death following loss of Par complex function

The death of early spermatocytes caused by loss of Par complex function in somatic cyst cells also required function of the small GTPase Rab35. Double knockdown of *rab35* with individual Par complex components partially rescued the survival of germ cells compared with double RNAi controls. As with the JNK pathway double RNAi rescue experiments, testes were classified according to the latest germ cell stage observed by phase-contrast microscopy. Again, adding a second control GFP RNAi line increased the percentage of testes with mature spermatocytes slightly for *aPKC* and *par6*, and more-so for *baz*. However, when Rab35 function was knocked down along with individual components of the Par complex, the majority (94.3-100%) of testes scored contained mature spermatocytes and nearly half (45.7-83.0%) of testes scored contained post-meiotic germ cells (Fig. 7A). Knockdown of Par complex transcripts was effective in the Rab35 double RNAi experiments, as shown by qrtPCR, in which knockdown of *baz*, *aPKC* or *par-6*, along with *rab35*, lowered Par complex transcript levels equally or more compared with transcript levels from testes in which a GFP RNAi construct was added instead of Rab35 (Fig. S4A-C).

Analysis of the nuclear Puc-LacZ reporter of JNK pathway activity indicated that Rab35 loss of function did not appear to rescue the Par loss-of-function phenotype by lowering JNK signaling pathway activity. Levels of Puc-LacZ were significantly increased by knockdown of aPKC, Par6 or Baz alone in both cyst cells associated with early spermatocytes (early SC CCs) (Fig. 7F) and in later Tj-negative cyst cells (late SC CCs) (Fig. 7G), as shown previously (Fig. 5E-G). Upon addition of a second RNAi construct, levels of the Puc-LacZ reporter in early SC CCs were not significantly higher than wild-type early SC CCs, potentially owing to lower levels of knockdown (Fig. 7F; Fig. S4). However, in late SC CCs, Par complex loss of function with a double RNAi control showed significantly higher levels of JNK pathway reporter activity compared with wild-type late SC CCs (Fig. 7B,C,G). Knockdown of *Rab35* under double RNAi conditions did not significantly lower levels of Puc-LacZ expression relative to double knockdown controls in either early or late SC CCs, suggesting that Rab35 may act downstream rather than upstream of activated JNK to enable cyst cells to kill neighboring spermatocytes (Fig. 7E-G). In contrast, analysis of expression levels of the Puc-LacZ reporter showed that knockdown of *bsk* under double RNAi conditions substantially lowered JNK pathway activity in late SC CCs in which Par complex components *aPKC* or *par6* had been knocked down relative to double RNAi controls (Fig. 7D,G).

## DISCUSSION

Our findings show that function of the Baz/Par-6/aPKC apical polarity complex in somatic cyst cells is required to suppress JNK pathway activation, and that aberrant JNK pathway activation due to loss of Par complex function causes the death of the early spermatocytes that the cyst cells enclose. The stage-specific death of spermatocytes observed requires action of the small GTPase Rab35 in cyst cells, suggesting participation of the recycling endosome in the mechanism that leads to non-autonomous germ cell death. This is reminiscent of somatic stretch follicle cells in the *Drosophila* ovary, which promote developmentally regulated stage-specific cell death of nurse cells prior to egg laying, a process that is dependent upon activation of the JNK pathway and intracellular trafficking proteins, including Rab35 (Timmons et al., 2016). We show that Rab35 acts either downstream or in parallel to JNK pathway activation in cyst cells that have lost Par complex function to kill neighboring spermatocytes.

Activation of the JNK pathway following loss of apical polarity in somatic cyst cells may be due to mechanisms similar to those seen in columnar epithelia in mammals or *Drosophila* imaginal discs, when Par complex function has been lost in a single cell or clone of cells in the epithelium (Archibald et al., 2015; Warner et al., 2010). In the fly wing imaginal disc, JNK signaling is linked to apical polarity loss through activation of Rho1 and phosphorylation of myosin by Rho kinase (Warner et al., 2010).

However, there are significant differences between the behavior of the squamous epithelial-like cyst cells that encapsulate male germ cell cysts and cells in traditional columnar epithelia. In columnar epithelial monolayers in *Drosophila* imaginal discs, loss of the Par complex initiates JNK pathway activation, which triggers cell-autonomous death of the depolarized cell and also triggers production of mitogenic signals that promote compensatory proliferation in neighboring cells in the epithelium (Fan and Bergmann, 2008; Warner et al., 2010; Ryoo et al., 2004; Kolahgar et al., 2015). In contrast, JNK activation following loss of Par complex function in somatic cyst cells in the *Drosophila* testis did not cause cyst cell death, but rather triggered non-autonomous killing of neighboring spermatocytes. It is possible that activation of the JNK pathway in cyst cells causes cyst cells to secrete a mitogenic signal that is received by the neighboring germ cells. However, because the spermatocytes are in a specialized extended G2 phase of meiosis I, they may be unable to respond appropriately to such pro-mitotic signals and instead undergo cell death (Fig. 7H,I).

The involvement of Rab35 in promoting death of spermatocytes following cyst cell depolarization suggests that trafficking of a signal to the cellular membrane may be required for the non-autonomous germ cell death. Rab35 is a small GTPase involved in rapid recycling of cargo from the early endosome back to the plasma membrane (Grant and Donaldson, 2009). Rab35 has been shown to be essential for recycling of the T-cell receptor (TCR) and its co-activators to the immunological synapse in cytotoxic T lymphocytes (CTLs) (Patino-Lopez et al., 2008), secretion of exosomes from glial cells (Hsu et al., 2010) and recycling of the yolk receptor in *C. elegans* oocytes (Sato et al., 2008). Because knockdown of Rab35 in cyst cells did not reduce activation of the JNK pathway in cyst cells but still partially prevented germ cell death when somatic cyst cells lose Par complex function, one possibility is that Rab35 function may be required to carry a product downstream of JNK pathway activation to the plasma membrane that faces the germ line, e.g. a pro-mitotic signal, as proposed above (Fig. 7I).

Strikingly, Par-complex function seemed to be less required in the squamous epithelial-like cyst cells than in columnar epithelia for formation of cell-cell junctions. Septate junctions normally form between the two cyst cells that surround each germ cell cyst and establish a permeability barrier by the four-cell spermatogonial cyst stage (Fairchild et al., 2015). Cyst cells appeared to maintain the ability to establish this permeability barrier under our conditions for knockdown of Baz, Par-6 or aPKC (Fig. 3F,G; Fig. S3). In addition, although adherens junction formation has been shown to depend on protein localization via the Par complex to establish an apical membrane domain in columnar epithelia (Müller and Wieschaus, 1996; Harris and Tepass, 2008; Baum and Georgiou, 2011), the death of spermatocytes after loss of Par complex function in cyst cells did not appear to result from destabilization of adherens junctions between cyst cells, as knockdown of E-cadherin in cyst cells did not result in spermatocyte death. In the *Drosophila* midgut, the Par complex is not required for establishment or maintenance of apical-basal polarity of epithelial enterocyte cells, which have a reversed arrangement of junctional complexes compared with epithelial cells of the *Drosophila* embryo or imaginal discs and are instead more similar to mammalian epithelial cell junctions (Chen et al., 2018). Instead, septate junction proteins and integrins are required to establish polarity and junction formation in the midgut epithelium (Chen et al., 2018). The decoupling of Par complex function from junctional stability in the cyst cells of the testis suggests that the cyst cell-cyst cell junction could also be established in a different manner from the cell-cell junctions in *Drosophila* epithelia in the wing disc or the embryo.

The stage-specific death of germ cells we observed, which was triggered non-autonomously upon loss of Par complex function in somatic cyst cells, resembles several physiological examples of germ cell death initiated by neighboring somatic cells. In *C. elegans*, DNA damage-dependent germ cell death is induced by an extracellular signal from the surrounding somatic intestine (Ito et al., 2010). Under starvation conditions, somatic cyst cells can trigger death and engulfment of early mitotic germ cells in the *Drosophila* testis, an event that has been proposed to protect the germline stem cell pool (Yang and Yamashita, 2015; Chiang et al., 2017). During *Drosophila* oogenesis, nurse cells, which manufacture most of the contents for the developing oocyte, eventually become acidified and engulfed by somatic stretch follicle cells (Timmons et al., 2017). Both nurse cell death and starvation-induced spermatogonial death require JNK pathway activation and activation of intracellular trafficking machinery for phagocytosis in the somatic cells that kill and engulf their neighbors (Etchegaray et al., 2012; Yang and Yamashita, 2015). The parallels between the mechanisms in place to eliminate nurse cells and our findings that cyst cells that have lost Par complex function initiate spermatocyte death raise the issue of whether loss of polarity in stretch follicle cells of the ovary precedes nurse cell death.

It is possible that cyst cells promote germ cell death as a consequence of Rab35 activity by engulfment of the spermatocytes, similar to other examples of somatic cell-induced germ cell death in the ovary and other organisms. In the ovarian follicle, stretch follicle cells engulf nurse cells through activity of an unknown engulfment receptor (Timmons et al., 2016). Perhaps Rab35 is involved in the recycling of a similar receptor in cyst cells in the testis? Rab35 is also required in macrophages for recruitment of proteins that rearrange the actin cytoskeleton in the early phagosome (Egami et al., 2011). Cyst cells that have lost the function of the Par complex may initiate actin cytoskelton rearrangments, dependent on the function of Rab35, to engulf neighboring spermatocytes (Fig. 7J).

Here, we advance two models by which loss of the Par polarity complex in somatic cyst cells of the *Drosophila* testis could lead to stage-specific germ cell death. In one, activation of the JNK pathway in cyst cells that have lost function of the Par complex is required for spermatocyte death, perhaps via production of a pro-death or pro-mitotic signal that is then carried to the plasma membrane that faces the germline by Rab35 (Fig. 7I). Another possibility is that Rab35 acts in parallel to signaling events downstream of JNK pathway activation, perhaps promoting phagocytosis of the dying spermatocytes by cyst cells that have lost function of the Par complex (Fig. 7J).

## MATERIALS AND METHODS

### Fly stocks and genetics

Flies were raised on standard cornmeal molasses agar medium. The following lines were used: *c587Gal4* (S. Hou, National Cancer Institute, Bethesda, MD, USA), Puc*-*LacZ (Glise et al., 1995), *Rbp4-eYFP* (Baker et al., 2015) and *bam-GFP* (Chen and McKearin, 2003). RNAi stocks were obtained from Vienna Drosophila Resource Center (VDRC): UAS *baz* RNAi (2914), UAS *aPKC* RNAi (105624 and 2907), UAS *par-6* RNAi (108560 and 19731), UAS *bsk* RNAi (31323) and UAS *Rab35* RNAi (28342). Stocks supplied from Bloomington Drosophila Stock Center were *baz::GFP* (51572), *shg::Tom* (58789) and UAS *gfp* RNAi (41558).

Acute knockdown by RNAi was performed using c587Gal4;;tubGal80ts crossed to UAS RNAi lines. Flies were raised at 22°C. Newly eclosed adult males were shifted to 30°C for 2-8 days and the phenotype was recorded at intervals throughout the time course.

### Immunostaining

For whole-mount immunofluorescence, testes were dissected in 1× PBS and fixed with 4% (vol/vol) formaldehyde in 1× PBS for 20 min at room temperature then washed twice with 1× PBS with 0.1% Triton-X 100 (PBST). Testes were permeabilized for 20 min in 1× PBS with 0.6% (vol/vol) Triton-100 and 0.6% (wt/vol) sodium deoxycholate at room temperature, then washed twice with PBST. Tissue was blocked overnight in 1× PBST with 3% (wt/vol) BSA at 4°C. Primary and secondary antibodies were diluted in 1× PBST with 3% (wt/vol) BSA. Tissue was incubated with primary antibodies for 48 h at 4°C and secondary antibodies for 2 h at room temperature away from light. Testes were washed twice with 1× PBST and mounted in Vectashield with DAPI (Vector Labs). For TUNEL assay, tissue was processed as described above, except that, after permeabilization, the protocol from the In Situ Cell Death Detection Kit (TMR Red, Sigma/Roche) was followed. For ring canal, fusome, Bam and Eya antibody staining, testes were flash frozen on slides and incubated for 10 min in 95% ethanol before fixation in 4% (vol/vol) formaldehyde in 1× PBS for 7 min at room temperature. Permeabilization was skipped and all incubation was carried out in a hydration chamber. Developmental Studies Hybridoma Bank (DSHB) supplied primary antibodies: mouse anti-FasIII (7G10; 1:10), mouse anti-Arm (N2 7A1; 1:10), mouse anti-Bam (1:10), mouse anti-Hts (1B1; 1:10), mouse anti-Eya (10H6; 1:10), mouse anti-Dlg (1:100) and mouse anti-Mmp1 (3B8, 3A6 and 5H7, used as a 1:1:1 mixture and then diluted 1:10). Santa Cruz Biotechnology supplied goat anti-Vasa (dC-13; 1:100). Other primary antibodies used were: rabbit anti-pTyr (Sigma 21315; 1:500), chicken anti-GFP (Abcam 13970; 1:10,000), rabbit anti-RFP (Rockland; 1:1000), mouse anti-βgal (Promega; 1:400), rabbit anti-Kmg (Kim et al., 2017; 1:400) and guinea pig anti-Tj (a gift from D. Godt, University of Toronto, Canada; 1:10,000). DyLight conjugated donkey secondary antibodies (Jackson ImmunoResearch Laboratories) were used at a concentration of 1:500.

### Live imaging

LysoTracker (ThermoFisher Red DND-99; final concentration of 50 ng/μl in PBS) was imaged live after 30 min incubation of testes at room temperature using a Leica SP8 confocal microscope as described by Yang and Yamashita (2015). The permeability assay was performed as described by Fairchild et al. (2015) with the exception that a 3 kDa AlexaFluor 680 Dextran (ThermoFisher) was used. For *baz::GFP* isolated cyst images, testes were dissected in 1× PBS, the sheath opened with forceps to spill out late stage cysts and imaged live.

### Image analysis and quantification

Images were taken using a Leica SP8 Confocal microscope and processed with ImageJ. Cells per cyst for counts of spermatogonial stages were equal to one less than the number of ring canals threaded by a fusome. To ensure that only early 16 cell cysts were counted, cysts with germ cells similarly sized to those of two-, four- or eight-cell spermatogonia were included in counts and cysts with larger germ cells were excluded. Nuclear *LacZ* quantification was carried out using ImageJ to acquire data from *z*-slices of confocal images to measure corrected total nuclear fluorescence [CTNF=Integreated Density−(Nuclear Area×Mean fluorescence of background readings)]. *LacZ* levels were measured from multiple cells (*n*>3) across multiple testes (*n*>4) for at least two biological replicates. No statistical methods were used to set sample size.

## Supplementary Material

Supplementary information
