# Supplementary material for: Somatic support cells regulate germ cell survival through the Baz/aPKC/Par6 complex
Source: Development. 2019 Apr 15;146(8):dev169342. doi: 10.1242/dev.169342 (PMC6503986; doi:10.1242/dev.169342)
Supplement: Supplementary information [file develop-146-169342-s1.pdf]

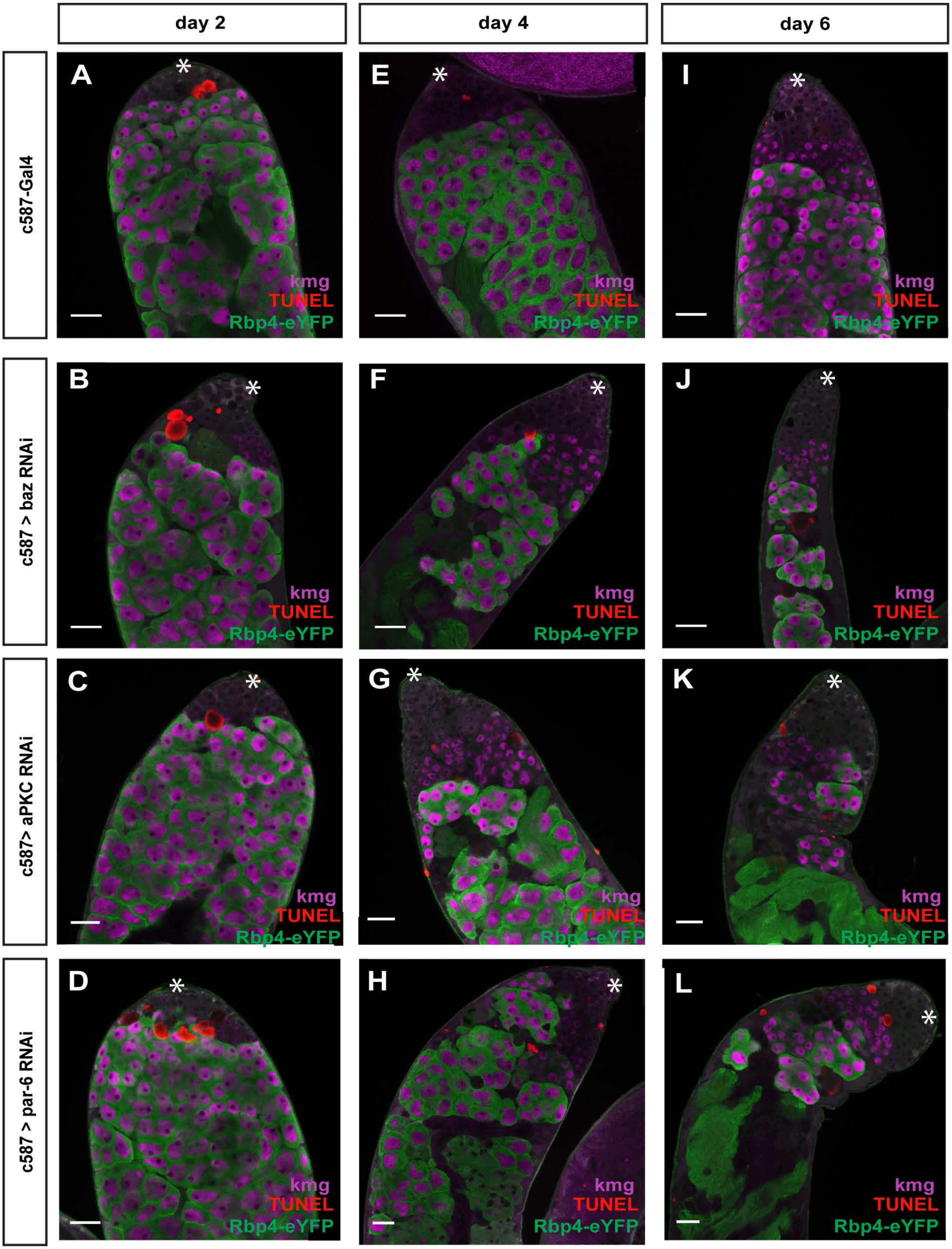

Development • Supplementary information

Figure S1. Progressive loss of early spermatocytes following cyst cell specific knockdown of Par complex proteins.

(A-L) Immunofluorescence images of testes assayed for TUNEL signal (red) from flies expressing Rbp4-eYFP 2 (A-D), 4 (E-H), and 6 (I-L) days after shift to 30 °C stained with anti-GFP (green) and anti-Kmg (magenta). Scale bar: 25 μm.

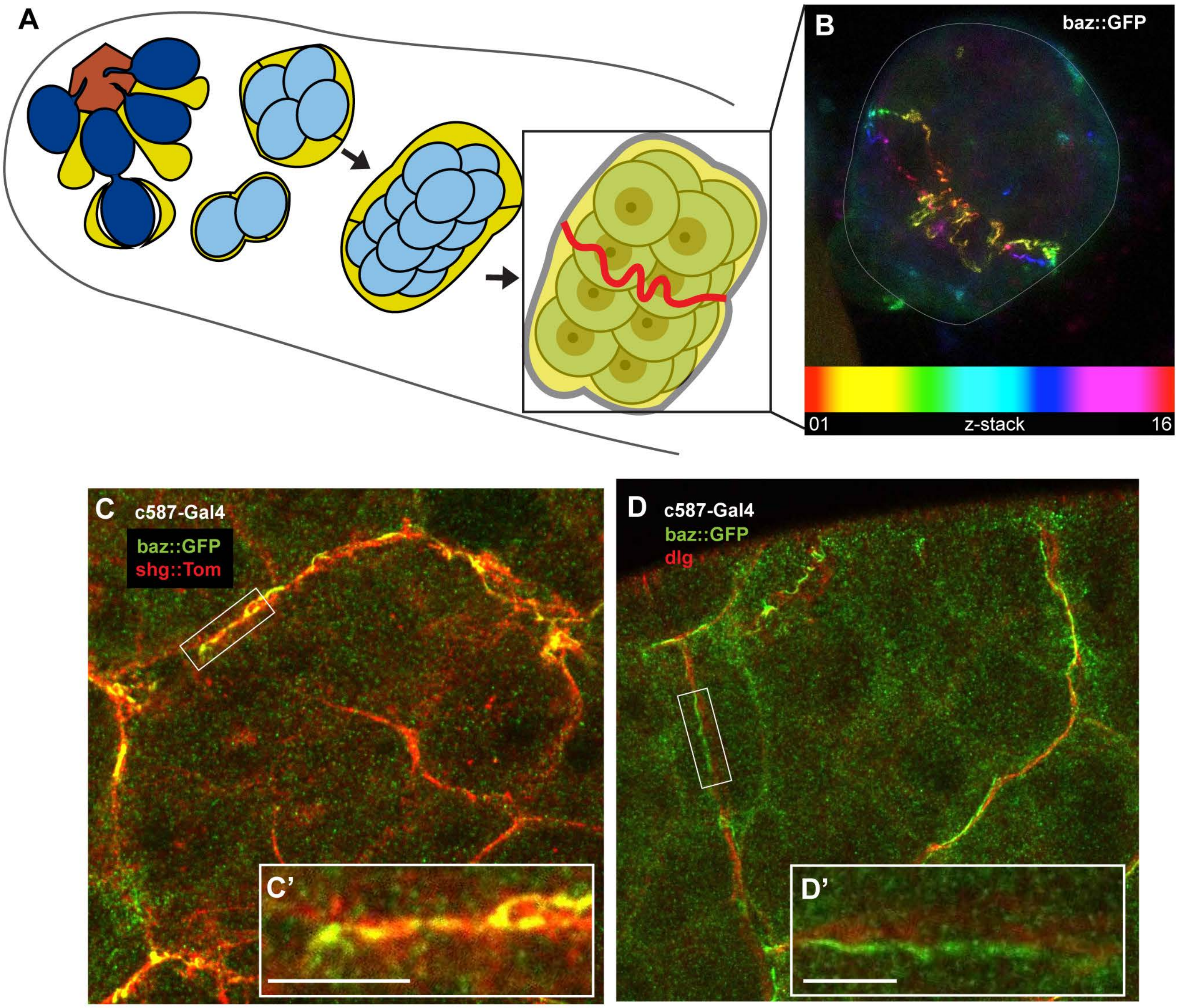

Figure S2. Cyst cells form stereotypical cell-cell junctions.

(A) Diagram of Drosophila spermatogenesis. Hub (orange); cyst cells (yellow); germline (blue); cyst cell-cyst cell junction (red).

(B) 3D projection of an isolated spermatocyte cyst from a testis from a fly expressing baz-GFP. Image is color-coded by z-stack. Scale bar: 12.5 μm.

(C) Immunofluorescence image of a spermatocyte cyst in a testis from a fly expressing baz-GFP and shg-Tomato stained with anti-GFP (green) and anti-RFP (red, adherens junction). Scale bar: 12.5 μm.

Development • Supplementary information

(D) Immunofluorescence image of spermatocyte cyst in a testis from a fly expressing baz-GFP stained with anti-GFP (green) and anti-Dlg (red, septate junction). Scale bar: 12.5 μm. (C’, D’) Higher magnification views of indicated areas in A and B. Scale bar: 6.25 μm.

Development • Supplementary information

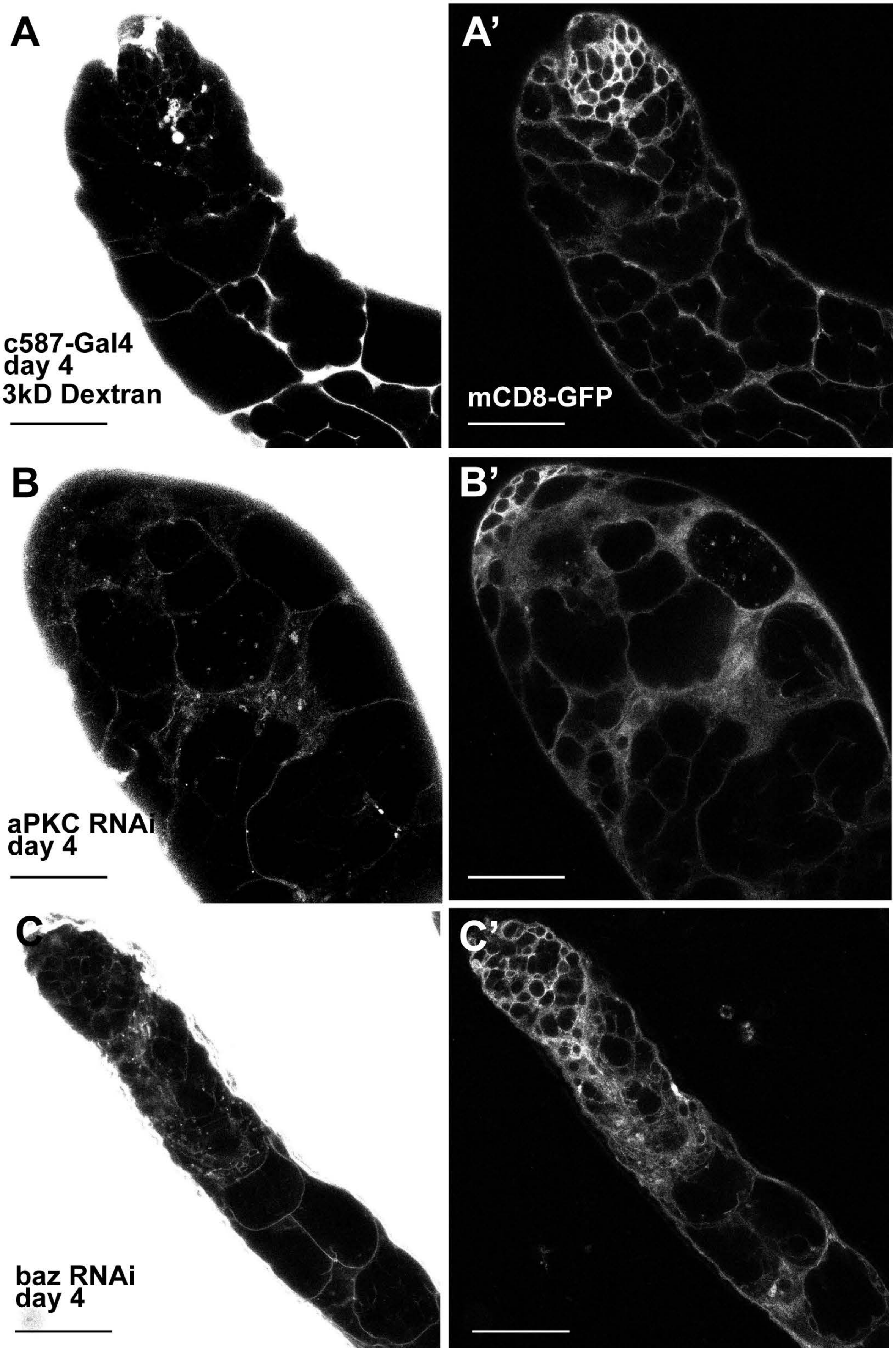

Development • Supplementary information

Figure S3. Cyst cells maintain a permeability barrier following Par complex knockdown.

(A-C) Images from permeability assays on live testes dissected from flies 4 days after shift to 30 °C. 3kD Dextran (A-C); mCD8-GFP (A'-C'). Scale bar: 50 μm.

Development • Supplementary information

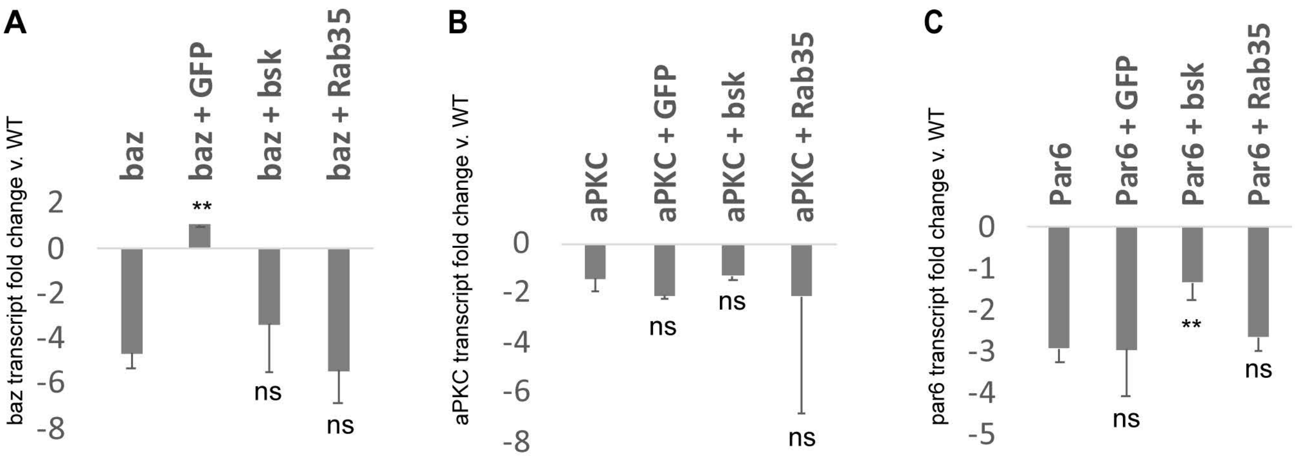

Figure S4. Par complex transcripts were efficiently knocked down in double RNAi rescue experiments.

(A-C) RT-qPCR of *baz* (F), *aPKC* (G), and *par6* (H) expression from testes of the indicated genotypes 8 days after shift to 30 °C. Data is shown as fold change relative to expression level in control testes. Significance determined by student's two-tailed t-test on DDCt values from two biological replicates (** indicates $p < 0.05$ compared to the single knockdown).
